# Trauma systems in Europe / hospital categories

**DOI:** 10.1007/s00068-025-02828-4

**Published:** 2025-03-19

**Authors:** Inger B. Schipper, Hans-Peter Simmen, Roman Pfeifer

**Affiliations:** 1 Department of Trauma Surgery, Leiden University Medical Center (LUMC), Leiden, The Netherlands; 2 Department for Traumatology, University Hospital Zurich, Zurich, Switzerland

**Keywords:** Polytrauma, Whitebook, ESTES

## Abstract

Trauma systems are vital components of healthcare infrastructure, addressing the significant burden of severe injuries across Europe. Effective trauma systems improve patient outcomes and reduce mortality by providing timely, specialised care. However, significant disparities remain between countries, with only a few well-structured and maintained systems currently operating in Europe.

Developing trauma systems requires collaboration among healthcare providers, emergency services, and government agencies. Standardised protocols for triage, transport, and treatment are essential, supported by robust infrastructure, public education, and injury prevention initiatives.

Trauma systems comprise four core components:

• Injury Prevention.

• Pre-Hospital Care.

• Facility Care.

• Post-Hospital Care/Rehabilitation.

These components rely on key elements such as leadership, professional resources, education, quality improvement, and funding. Political commitment, geographical considerations, and the efforts of dedicated clinicians are crucial for ensuring system success.

Trauma systems across Europe are evolving under diverse healthcare structures. Over recent decades, dedicated clinicians, often with support from national medical societies, have initiated and sustained these systems. Typically, trauma hospitals, or trauma centres (TCs), are categorised into two or three levels, with the highest being ‘Level I TC’ or ‘Major TC,’ capable of managing the most complex cases. This chapter outlines general requirements for these categories, leaving individual nations to tailor standards to their healthcare systems.

## Introduction

The need for trauma systems in Europe has grown steadily with the rising incidence of traumatic injuries. This has coincided with increasing sub-specialisation of healthcare personnel and reductions in working hours. Traffic accidents, occupational incidents, and incidents related to violence contribute significantly to the burden of trauma cases across the region. Studies have shown that the timely and appropriate delivery of trauma care can significantly impact patient survival rates and long-term functional outcomes.

Following the implementation of an inclusive trauma system in the United States, Europe has been slower to adopt similar measures. In many European countries, trauma systems have not been designed and developed based on international standards and quality guidelines, but instead reflect historical, geographical, and demographic circumstances. Although there is no universal gold standard for trauma systems, generic elements remain consistent, and guidelines have been developed that apply across all countries.

## Essential components and infrastructure of trauma systems

The organisation of trauma systems is a critical aspect of providing effective and comprehensive care to individuals with traumatic injuries. The American College of Surgeons (ACS) and the American Association for the Surgery of Trauma (AAST) have outlined a framework for trauma systems with the four fundamental components mentioned above.

Within these four fundamental components, the following elements should be considered: Leadership, Professional Resources, Infrastructure, Education and Advocacy, Information, Finances, Quality Improvement, Research, Technology, and Disaster Preparedness and Response. Each of these elements has specific requirements that must be fulfilled and maintained.

### Injury prevention

Injury prevention aims to reduce the occurrence of traumatic injuries through educational initiatives, safety campaigns, and policy development. This component focuses on minimising the risk of injuries in the first place. The infrastructure elements within this component may include:

- **Leadership**: Appoint leaders or committees for injury prevention.
- **Professional Resources**: Engage experts in injury prevention strategies.
- **Education and Advocacy**: Promote public education and safety policies.
- **Information**: Use injury data to guide prevention efforts.
- **Finances**: Secure funding for prevention programmes.

### Pre-hospital care

This component involves providing rapid and appropriate care at the scene of the injury and during transportation to a pre-defined healthcare facility. Infrastructure elements within pre-hospital care may include:

- **Leadership**: Establish roles to coordinate pre-hospital care services.
- **Professional Resources**: Ensure trained first responders (e.g., paramedics, EMTs).
- **Education and Advocacy**: Train responders in triage and transport protocols.
- **Technology and Infrastructure**: Equip responders with assessment and communication tools.
- **Information**: Establish communication systems for patient handover.

### Facility-based care

Facility-based care involves the treatment and management of traumatic injuries in healthcare facilities, ranging from dedicated TCs (trauma center) to general hospitals with trauma care capabilities and rehabilitation centres. Hospitals should be categorised to clearly define their roles and responsibilities. Determining how many major TCs are needed for a specific population and how the network around the TCs should be organised remains a task that has not been undertaken in many European countries.

- **Leadership**: Designating leadership roles to oversee trauma care delivery at all hospital levels, including the Emergency Department (ED) trauma team and inpatient services.
- **Professional Resources**: Assembling skilled trauma teams and specialists including trauma surgeons, nurses, anaesthesiologists, and other specialists. Dedicated TCs must provide acute and critical care, surgical interventions, definitive management and multidisciplinary rehabilitation services. They need a dedicated trauma service coordinating multidisciplinary care throughout the hospital stay.
- **Education and Advocacy**: Providing ongoing training based on predefined competency goals for all personnel involved, focusing on the latest trauma care techniques.
- **Technology and Infrastructure**: Ensuring minimum equipment requirements for hospitals certified to treat trauma patients.
- **Information**: Establishing systems to share patient information and treatment protocols among different healthcare facilities.
- **Quality Improvement**: Implementing systems that use data and self-evaluations for continuous care assessment and quality assurance.
- **Finances**: Allocating resources to maintain well-equipped major TCs and support specialised staff, with clearly defined requirements for hospitals treating trauma patients.

### Post hospital Care / Rehabilitation

This component focuses on the long-term recovery and rehabilitation of trauma survivors after discharge from the healthcare facilities. Infrastructure elements within post-hospital care include:

- **Leadership**: Identifying or establishing roles to coordinate post-hospital care services.
- **Professional Resources**: Connecting patients with appropriate rehabilitation specialists, counsellors, and support groups.
- **Education and Advocacy**: Raising awareness of post-hospital care and resources.
- **Information**: Sharing patient progress and treatment plans among healthcare providers.
- **Quality Improvement**: Collecting data and self-evaluations for internal quality assurance.
- **Finances**: Securing funding for rehabilitation services and ongoing support.

Overall, the organisation of trauma systems based on these four fundamental components and their corresponding infrastructure elements helps ensure a coordinated and comprehensive approach to trauma care. To effectively manage trauma cases, trauma systems in Europe should work collaboratively to implement them.

## Generic needs for an inclusive trauma system

- **Communication Systems**: Effective communication networks between EMS providers, trauma centres, and other healthcare facilities are needed to transmit vital information about the patient’s condition. These networks facilitate timely decision-making and coordinate resources for optimal patient care.
- **Trauma Registries**: Trauma registries are databases that collect and store detailed information on trauma cases, treatment outcomes, and long-term follow-up data. These registries are used to assess the effectiveness of trauma systems, identify areas for improvement, and conduct research to enhance trauma care practices.
- **Continuous Quality Improvement**: Regular evaluation and improvement of trauma care processes through quality improvement initiatives enhance the overall trauma system performance. This involves analysing outcomes (e.g., data audits and mortality/morbidity meetings), identifying trends, and implementing evidence-based best practices to optimise patient care.

## The influence of politics and geography

The establishment and functioning of trauma systems in Europe are significantly influenced by political factors operating at national, regional, and local levels. Political commitment and support, in addition to dedicated medical professionals, are needed to develop and sustain trauma systems. Adequate funding ensures the availability of resources, staffing, and infrastructure required to deliver high-quality trauma care. Moreover, legislation and policies related to trauma care, such as seatbelt laws, traffic regulations, and workplace safety standards, can significantly impact the incidence and severity of traumatic injuries.

Geographical factors also play a significant role in shaping European trauma systems. Diverse topography, population distribution, and transportation networks affect the accessibility and availability of trauma care in different regions. Rural and remote areas may face challenges in ensuring timely access to trauma hospitals, necessitating strategies for efficient pre-hospital care and inter-facility transfers. Regions with high traffic density or those prone to specific types of injuries may require tailored approaches to trauma system organisation and resource allocation.

## Conclusion and needs for the future

Trauma systems in Europe are essential for addressing the growing burden of severe injuries and providing timely and specialised care to trauma victims. The coordination of designated trauma centres, efficient triage and transport protocols, robust communication systems, trauma registries, and continuous quality improvement initiatives form the foundation of these systems. Effective organisation, political commitment, and geographical considerations are crucial for the successful implementation and sustainability of trauma systems across the diverse European landscape. Given the heterogeneity of European healthcare systems, specific adaptations must be made for each country (Fig 1).

## Hospital categories and their roles in the trauma system

**Fig. 1:**
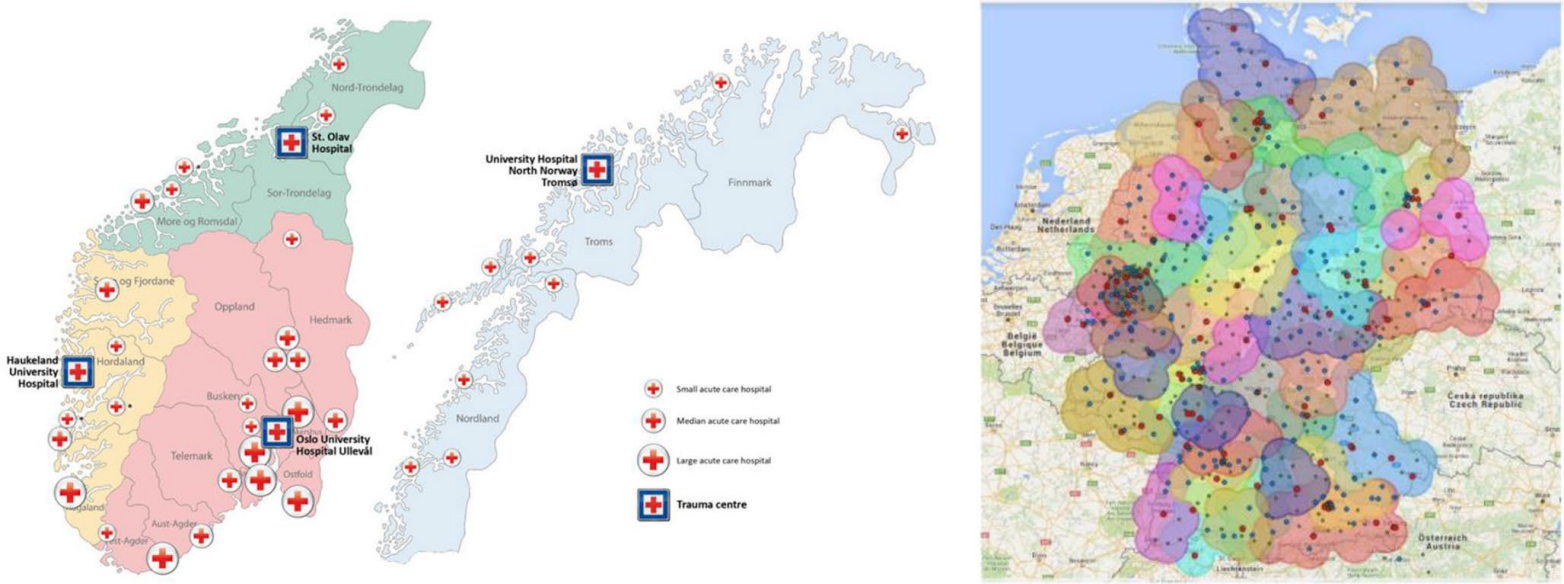
Trauma Networks (**a**) Norway and (**b**) Germany. Note: Naming conventions for trauma hospitals vary across countries. In this document, ‘Level I TC,’ ‘Level II TC,’ and ‘Level III TC’ are used generically to describe maximum, intermediate, and basic levels of trauma care. Equivalent terms, such as “major trauma centres” or “trauma units,” may apply depending on the national healthcare system

## Introduction

Trauma hospital classification ensures optimal resource allocation and patient care. The benefits include:

- **Severity-Based Categorisation**: Patients are referred to facilities best equipped for their injuries.
- **Efficient Resource Allocation**: Aligns hospital resources with patient needs.
- **Improved Field Triage**: Guides EMS to transport patients to the nearest appropriate facility.
- **Multidisciplinary Care**: Higher-level centres offer specialised expertise for complex injuries.
- **Continuous Improvement**: Training and research at TCs advance trauma care standards.
- **Guideline Compliance**: Hospitals meet care standards, including transfer protocols.
- **Mass Casualty Management**: Higher-level centres are better equipped for large-scale emergencies.

## General descriptions of hospital levels

The descriptions below provide a generic overview of trauma-receiving facilities, as each country must adjust requirements to its specific healthcare system. For detailed descriptions, refer to examples from the German Trauma Society, the updated UK NICE guidelines, the American College of Surgeons (ACS) guidelines, and national system descriptions developed by some European countries.

*In general*,* hospitals lacking essential 24/7 infrastructure should not be designated for trauma patient care.*

### Maximum level of care (Major TC or level I TC)

Major TCs (often university hospitals) are responsible for the comprehensive care of multiple and severe injuries. In addition, these centres treat patients with exceptionally complex or rare injury patterns. A dedicated trauma service is mandatory, with all specialties available. These TCs must have intensive care and surgical capacity for immediate admission at all times. Clear criteria should be established for transferring patients from lower-level trauma hospitals to the highest level of care within the network.

## Medium level of care (Level II TC)

Medium-level trauma hospitals provide comprehensive emergency and definitive care for severely injured patients. Key elements include the permanent presence of specialists trained in trauma care and access to consultants from other disciplines (e.g., neurosurgery). These hospitals must also have adequate diagnostic, therapeutic, and surgical equipment. Medium-level trauma hospitals should be capable of managing the majority of injuries and their sequelae with definitive care.

## Minimum level of care (Level III TC, trauma units, or emergency Hospitals)

The primary role of these hospitals is to provide care for common isolated injuries. In addition, these centres serve as the first point of contact, especially in rural areas, providing appropriate emergency care, including Damage Control Surgery (DCS), resuscitation, and referral of severely injured patients to higher levels of care. Treating life-threatening conditions and ensuring transport to the nearest appropriate trauma centre are the primary responsibilities of these facilities.

## Levels of care (summary)

In short, the levels of care are defined as follows:

- **Level I (Maximum Care)**: Comprehensive care for multiple, severe, and complex injuries with 24/7 dedicated trauma service and specialty coverage from all involved specialities.
- **Level II (Intermediate Care)**: Full emergency and definitive care capabilities, including consultant access and multidisciplinary expertise.
- **Level III (Basic Care)**: First-line care for isolated or life-threatening injuries, focusing on stabilization and referral.

## Conclusion and needs for the future

The diversity in European healthcare systems necessitates tailored approaches to trauma care. Future efforts should prioritize integrating trauma networks, fostering collaboration among all levels of care, and developing active systems where none exist. Such initiatives will ensure equitable, high-quality trauma care across regions.

## Data Availability

No datasets were generated or analysed during the current study.
